# Editorial: Human brain organoids to model neurodegenerative diseases at the BOSS23 Brain Organoid Summer School

**DOI:** 10.3389/fncel.2024.1501036

**Published:** 2024-11-05

**Authors:** Fabio Cavaliere, Dirk M. Hermann, Chiara Magliaro

**Affiliations:** ^1^Achucarro Basque Center for Neuroscience, The Basque Biomodels Platform for Human Research, Leioa, Spain; ^2^Fundación Biofisica Bizkaia, Leioa, Spain; ^3^Department of Neurology, University Hospital Essen, University of Duisburg-Essen, Essen, Germany; ^4^Centro di Ricerca “E. Piaggio”, University of Pisa, Pisa, Italy; ^5^Department of Information Engineering, University of Pisa, Pisa, Italy

**Keywords:** brain organoids, neurodegenerative diseases, Amyotrophic lateral sclerosis, Parkinson's disease, Alzheimer's disease

Brain organoids hold great promise in recapitulating human neurological diseases, which might help overcoming limitations in the translation of research findings into clinical progress. However, while brain organoids effectively recapitulate key developmental stages of the human brain, their use in studying the onset and mechanisms of neurodegenerative diseases (NDs) still faces significant challenges.

To this end, in June 2023, the first International Brain Organoid Summer School (BOSS23) was organized by the Basque Biomodel Platform for Human Research (BBioH) at the Achucarro Basque Center of Neuroscience (Bilbao, Spain). BOSS23 provided an unique opportunity for young inspired researchers to get in touch with leading experts in the field to discuss on the potential of human brain organoids to model age-dependent NDs. After the Summer School, we invited participants of this conference to provide contributions to this Research Topic collection.

The use of brain organoids as a model to study age-related NDs is still in its infancy, making brain organoid research an exciting field of investigation. The current challenges of brain organoids were extensively discussed in a review by Urrestizala-Arenaza et al., who identified the absence of microglia and vasculature as major obstacles in studying neurodegenerative diseases (NDs). The authors focused on Alzheimer's disease, Parkinson's disease, and amyotrophic lateral sclerosis (ALS), where neuroinflammation and impaired neurovasculature are key features of neurodegeneration. They specifically highlighted structural and biological limitations, such as the lack of an aging signature, angiogenesis, and myelination, as significant drawbacks in using brain organoids to model age-related NDs.

The presence of immature structures was demonstrated in the original article by Mateos-Martínez et al.. In their contribution, the authors provided insights into the morphological and ultrastructural composition of mature brain organoids. Their work supported the hypothesis that brain organoids have strong prospects, but in their current form still have limitations for studying age-related NDs. The development of proliferative zones in brain organoids was remarkably similar to those found in human brain development, with cells exhibiting polarized structures surrounding a central cavity with tight junctions and cilia.

Nevertheless, brain organoids remain a valuable model for studying neural metabolism and related pathologies, particularly those involving mitochondria and neuronal circuitry. Coronel et al. emphasized in their review that brain organoids provide a useful platform for investigating cellular metabolism and neuronal communication at various stages of development as well as in NDs. Mitochondria are central regulators of neurogenesis and many pathologies of the central nervous system. Diseases caused by alterations in mitochondrial DNA (mtDNA) and nuclear DNA (nDNA) have proven challenging to model due to their complexity. However, recent advancements in methodologies applied to brain organoids have created new opportunities to develop innovative models for brain metabolism studies, as the authors pointed out.

Heesen and Köhr reviewed the importance of GABAergic interneurons in brain network function using iPSC-derived brain organoids. The three-dimensional structure offered by 3D models highlighted the role of GABAergic signaling in human-specific brain networks, suggesting that the cortical organization of GABAergic interneurons in 3D is essential for generating synchronous neuronal firing patterns.

Finally, an intriguing perspective was presented on the use of brain organoids to explore the heart-brain axis by Mabry and Pavon, as seen in some rare NDs, expanding the potential applications of these models beyond the central nervous system (CNS). The organoid model may serve as an advanced tool for studying genetic forms of Parkinson's disease, such as those caused by the rare E46K mutation in alpha-synuclein, as well as for modeling sympathetic cardiomyocyte regulation during the early fetal period.

The works in this Research Topic show that, despite the current limitations, brain organoids have become a valuable model for studying neural metabolism and related pathologies. Brain organoids offer a useful platform for investigating cellular metabolism and neuronal communication. However, technical challenges such as heterogeneity in size and morphology, handling difficulties, and scalability still limit their application under conditions of NDs. Existing limitations must be overcome, e.g., by incorporating additional cell types into the organoid models. Some of these limitations could be addressed by standardizing protocols, adopting engineering solutions, automating handling, and utilizing high-content analysis. The field is eagerly waiting for a follow-up conference.

